# The Powers of the Ship's Surgeon

**Published:** 1895-09-28

**Authors:** 


### Editor's Letter-Box.

[Our correspondents are reminded that proxility is a great bar to publication, and that brevity of style and conciseness of statement greatly facilitate early insertion.]

### THE POWERS OF THE SHIP'S SURGEON.

Dr. Leet writes: In your issue of September 14th, annotation on this subject, you quote from the title-page of my pamphlet, "Ship Owners and Ship Surgeons," viz.: "The author's services were dispensed with by three of the foremost shipping companies in the world after conscientiously discharging his duty in these three passenger lines, and with satisfactory testimonials from each company." You proceed thus: "Moreover, now that Dr. Leet has settled down to practise on shore, the employés of these companies have been warned that it will be injudicious for them to employ him. This is a serious charge, and we regret that Dr. Leet has not thought it wise to substantiate it by naming the companies. . . . We have no sympathy with anonymous accusations. Let Dr. Leet name the criminals, and he may be sure of both support and sympathy." Will you kindly insert for the perusal of your readers the whole paragraph, when it will be seen that I made no charge or accusation. Here is the cutting from the pamphlet, p. 8:—

Dr. Leet regrets that, through fear of giving offence to shipowners—their employers, the officers, engineers, and chief stewards dare not employ his professional services, and are probably expected to warn all their seamen, sailors, firemen, stewards, cooks, butchers, emigrants, and shore employés to give Dr. Leet "a wide berth," for having had the courage and conscience to shock the public by exposing the infractions as well as evasions of the British Passenger Acts and Her Majesty's Board of Trade Orders in the Atlantic passenger trade, and the ship surgeons' neglect of exposing their degraded position all these years past until Dr. Leet set them the example, and was awarded by the steamship managers the penalty (*shirked by them*), namely, "*your services are dispensed with.*" *Truth* correctly described this penalty in its admirable article.

There is a *lex non scripta* in the mercantile navy to the effect that any official serving afloat who writes to the papers or publishes facts *re* insanitary conditions on board his ship may expect dismissal at the end of any voyage without a day's notice or a day's pay, and any member of a ship's company who maintains acquaintance with the dismissed one, be he engineer, deck officer, or doctor, incurs the displeasure of the managers and the shore heads of departments. I published nothing until I settled down to practise on shore in 1889, when my "Ship Surgeon of To-day" was published, containing names of the companies and official letters to H.M. Board of Trade, reporting the abuses, &c., alluded to above, amongst them the following serious order of the Cunard Company. For four years previous to my appointment to that company the managers had forbidden their medical officers in the Atlantic passenger fleet to fill up their monthly sanitary reports for H.M. Board of Trade according to the Board's printed official instructions, which required special mention of the condition of the ventilation, any disinfection of the ship after contagious disease, and complaints of emigrants during the voyage. These three subjects of vital importance were deliberately omitted from our report, consequently the managers resented my exposure of such conduct in my official report to the Board of Trade, which also appeared in the *British Medical Journal*, November, 1889. The employés of the Cunard Company boycotted me ever since for not having shut my eyes to all abuses.

⁂ Dr. Leet has gratified us by naming at least one company in which the Board of Trade regulations are evaded. If the ship surgeons of the Cunard fleet are forbidden to fill up their report in full it is for the officers of the Board of Trade to note the omission and inquire the cause of it. If Dr. Leet has brought to light any unworthy connivance between the shipping company and the Board of Trade officials he has done well, and all honest people must thank him for it. At the same time one must recognise the difficulty of making a floating hospital sanitarily perfect. Space is limited, and ventilation must be more or less artificial. The writer of the annotation had investigated the hospital of an Atlantic liner. From a shore point of view it was bad, but compared with the best accommodation given to any one on the ship it was by no means bad. This was only a second-rate vessel, and the writer regrets that he has not been privileged to visit the hospital on board any Cunarder—passengers are not allowed to go where they have no business in that fleet. But, having crossed in the "Lucania," he is prepared to say that to be ill even in a good state-room on that best of boats would be no slight trial. In short, sickness at sea is, at best, accompanied by much more disadvantageous circumstances than sickness on land; many of the disagreeables are inevitable, and are recognised as such by the surgeons; hence, very little is said about them. As for the others, it is for ship surgeons themselves to state them in their reports. Only by their own loyalty to their trust, by their fearless honesty, can they raise themselves from their "degraded position." "Who would be free, themselves must strike the blow!" True, they may—at least the first one or two of them—reap the reward of "having their services dispensed with"; but, at the best, the remuneration and conditions attached to medical service in the merchant marine are not such as to make it desirable, as a permanent appointment, to really good doctors. Many smart young men take these posts as being a cheap and pleasant way of seeing the world, but few grow old at the work. Therefore few, and these few very rarely of the best, need hesitate to tell the plain, unpalatable truth concerning abuses on board ship. They have little to lose; and we do not think so meanly of the medical profession as to think that they cling so much to their small salary that they will lie and equivocate and cringe in order to keep it for a few months longer. At least, shore doctors are not like that. And we do not believe that the Cunard or any other company, catering as it does for public support, dare face a public exposure of genuinely insanitary conditions and hospital accommodation.—Ed. *T. H.*

